# *N*,*N*-Dimethyl-4-(pyren-1-yl)aniline

**DOI:** 10.1107/S1600536813032698

**Published:** 2013-12-07

**Authors:** Sreevidya Thekku Veedu, Mirko Scholz, Reza Kia, Carsten Paulmann, Simone Techert

**Affiliations:** a FS–SCS, Deutsches Elecktronen-Synchrotron (DESY), Notkestrasse 85, 22607 Hamburg, Germany; b Max Planck Institute for Biophysical Chemistry, Am Fassberg 11, 37077 Göttingen, Germany; c FS–PS, Deutsches Elecktronen-Synchrotron (DESY), Notkestrasse 85, 22607 Hamburg, Germany

## Abstract

In the title compound, C_24_H_19_N, the di­methyl­amino group is inclined to the benzene ring by 2.81 (9)°. Their mean plane makes a dihedral angle of 64.12 (2)° with the mean plane of the pyrene ring system [r.m.s. deviation = 0.031 (1) Å]. In the crystal, mol­ecules are linked *via* C—H⋯π inter­actions, which connect neighbouring mol­ecules into columns along the *c* axis.

## Related literature   

For charge transfer involving donor and acceptor mol­ecules, see: Wasielewski (1992 ▶); Willemse *et al.* (2000 ▶). For a related structure, *N,N*-Diphenyl-4-(pyren-1-yl)aniline, see: Wang *et al.* (2010 ▶). For the synthesis of the title compound, see: Dewar & Mole (1956 ▶); Norman *et al.* (1958 ▶). For standard bond lengths, see. Allen *et al.* (1987 ▶).
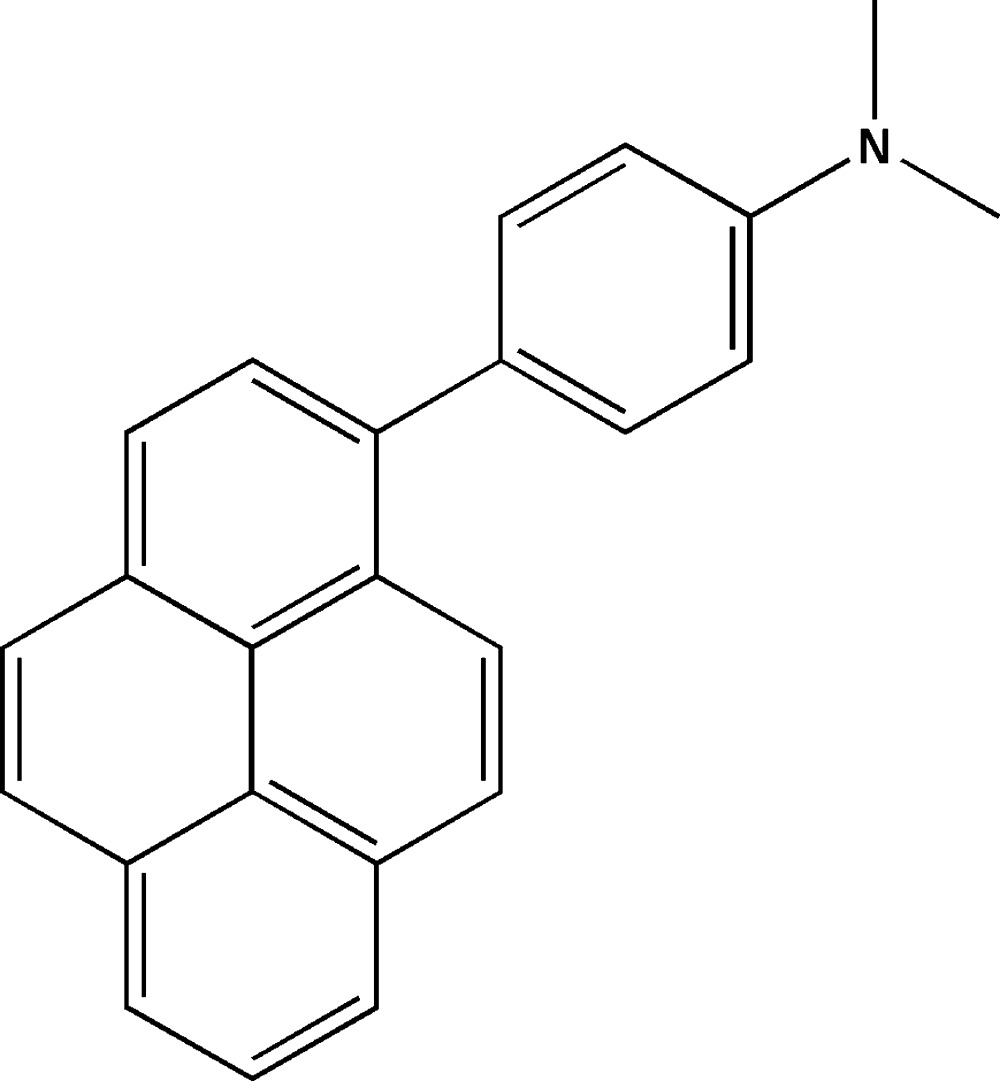


## Experimental   

### 

#### Crystal data  

C_24_H_19_N
*M* *_r_* = 321.40
Monoclinic, 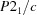

*a* = 6.1270 (12) Å
*b* = 30.686 (6) Å
*c* = 9.478 (3) Å
β = 113.35 (2)°
*V* = 1636.0 (8) Å^3^
*Z* = 4
Synchrotron radiation
λ = 0.600 Å
μ = 0.08 mm^−1^
*T* = 100 K
0.30 × 0.15 × 0.10 mm

#### Data collection  

Huber diffractometer with a Mar CCD detector
Absorption correction: multi-scan (*SADABS*; Bruker, 2005 ▶) *T*_min_ = 0.978, *T*_max_ = 0.993
47866 measured reflections
5872 independent reflections
4806 reflections with *I* > 2σ(*I*)
*R*_int_ = 0.063

#### Refinement  

*R*[*F* ^2^ > 2σ(*F* ^2^)] = 0.047
*wR*(*F* ^2^) = 0.135
*S* = 1.08
5872 reflections
228 parameters
H-atom parameters constrained
Δρ_max_ = 0.40 e Å^−3^
Δρ_min_ = −0.23 e Å^−3^

### 

Data collection: *XDS* (Kabsch, 1993 ▶); cell refinement: *XDS*; data reduction: *XDS*; program(s) used to solve structure: *SHELXS97* (Sheldrick, 2008 ▶); program(s) used to refine structure: *SHELXL2013* (Sheldrick, 2008 ▶); molecular graphics: *SHELXTL* (Sheldrick, 2008 ▶); software used to prepare material for publication: *PLATON* (Spek, 2009 ▶).

## Supplementary Material

Crystal structure: contains datablock(s) global, I. DOI: 10.1107/S1600536813032698/su2671sup1.cif

Structure factors: contains datablock(s) I. DOI: 10.1107/S1600536813032698/su2671Isup2.hkl

Supporting information file. DOI: 10.1107/S1600536813032698/su2671Isup3.cml

Additional supporting information:  crystallographic information; 3D view; checkCIF report

## Figures and Tables

**Table 1:** Hydrogen-bond geometry (Å, °) *Cg*1, *Cg*2, *Cg*3, *Cg*4 and *Cg*5 are the centroids of the C1–C6, C7–C10/C19/C20, C10–C13/C18/C19, C13–C18 and C17–C22 rings, respectively.

*D*—H⋯*A*	*D*—H	H⋯*A*	*D*⋯*A*	*D*—H⋯*A*
C2—H2⋯*Cg*2^i^	0.95	2.93	3.6043 (15)	129
C6—H6⋯*Cg*5^ii^	0.95	2.90	3.6495 (15)	137
C22—H22⋯*Cg*1^iii^	0.95	2.68	3.5499 (15)	152
C23—H52*A*⋯*Cg*3^i^	0.98	2.74	3.5994 (15)	147
C24—H52*E*⋯*Cg*4^ii^	0.98	2.77	3.5284 (15)	135

Symmetry codes: (i) 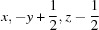; (ii) 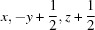; (iii) 
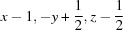
.

**Fig. 1.**
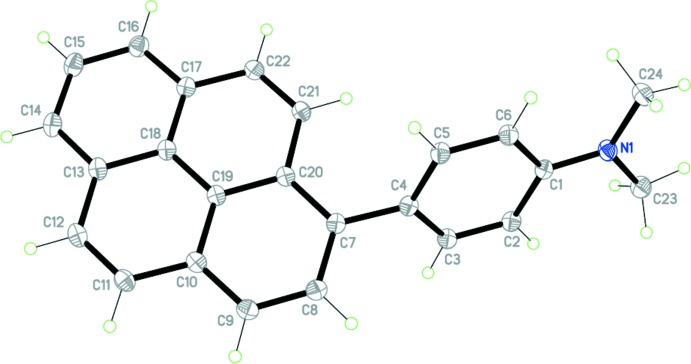
The molecular structure of the title molecule, with atom labelling. Displacement ellipsoids are drawn at the 50% probability level.

**Fig. 2.**
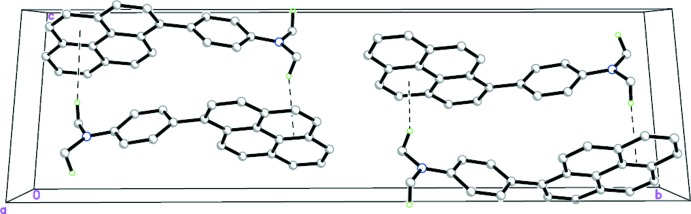
The crystal packing of the title compound, viewed along the a axis, showing molecules linked by C—H···π interactions (dashed lines; see Table 1 for details).

## supplementary crystallographic information

### 1. Comment

Electron donor acceptor molecules play an important role in the understanding of charge transfer processes. In the past decades, in order to gain more insight into electron transfer processes, extensive studies have been carried out on the optical behavior of systems consisting of donor acceptor groups linked by different bridges (Wasielewski, 1992; Willemse *et al.*, 2000). These molecules are also ideal systems for studying the solvation dynamics and also for the demonstration of non-linear optical properties. Pyrene-*N,N*-dimethylaniline (PyDMA), is a compound in which the electron donor *N,N*-dimethylaniline (DMA) is covalently linked to the electron acceptor pyrene. Due to the lack of an extended bridge between the donor and acceptor in PyDMA, the physical characteristics of these groups strongly influence the electron transfer mechanism. This leads to a very unusual absorption and emission spectra in the optical regime and because of this PyDMA is considered to be a molecular diode where electron donor and electron acceptor moieties are twisted against each other modulating the electron charge transfer processes.

The title compound, Fig. 1, is a pyrene derivative. The bond lengths (Allen *et al.*, 1987) and angles are within the normal ranges and are comparable to those reported for a similar structure, *N,N*-Diphenyl-4-(pyren-1-yl)aniline (Wang *et al.*, 2010). The dimethylamine group and the benzene ring are almost coplanar (dihedral angle = 2.81 (9) °) and their mean plane makes a dihedral angle of 64.12 (2) ° with the pyrene ring system [r.m.s.d. = 0.031 (1) Å].

In the crystal, packing is stabilized by C—H···π interactions (Table 1). The interaction C23—H52A···*Cg*3^i^ (see Table 1; *Cg*3 is the centroid of the C10-C13/C18/C19 ring) connects neighbouring molecules into columns along the *c*-axis (Fig. 2).

### 2. Experimental

Commercially available 1-aminopyrene after diazotization reaction was coupled with *N,N*-dimethylaniline according to the previously reported procedure (Dewar & Mole, 1956; Norman *et al.*, 1958). The crude product was then purified on an aluminium oxide column with a mixture of cyclohexane/toluene as eluent and applying HPLC. Block-like colourless crystals of the title compound were obtained by crystallization from ethyl acetate/diethyl ether (2:1) by slow evaporation.

### 3. Refinement

The C-bound H-atoms were included in calculated positions and treated as riding atoms: C—H = 0.95 and 0.98 Å for CH and CH_3_ H-atoms, respectively, with *U*_iso_(H) = 1.5*U*_eq_(C-methyl) and = 1.2U_eq_(C) for other H atoms.

### Crystal data

**Table:**

C_24_H_19_N	*F*(000) = 680
*M**_r_* = 321.40	*D*_x_ = 1.305 Mg m^−^^3^
Monoclinic, *P*2_1_/*c*	Synchrotron radiation, λ = 0.600 Å
*a* = 6.1270 (12) Å	Cell parameters from 2549 reflections
*b* = 30.686 (6) Å	θ = 2.5–26.7°
*c* = 9.478 (3) Å	µ = 0.08 mm^−^^1^
β = 113.35 (2)°	*T* = 100 K
*V* = 1636.0 (8) Å^3^	Block, colourless
*Z* = 4	0.30 × 0.15 × 0.10 mm

### Data collection

**Table:**

Huber diffractometer with a Mar CCD detector	4806 reflections with *I* > 2σ(*I*)
Radiation source: synchrotron	*R*_int_ = 0.063
φ and ω scan	θ_max_ = 27.0°, θ_min_ = 2.1°
Absorption correction: multi-scan (*SADABS*; Bruker, 2005)	*h* = −8→9
*T*_min_ = 0.978, *T*_max_ = 0.993	*k* = −44→45
47866 measured reflections	*l* = −14→14
5872 independent reflections	

### Refinement

**Table:**

Refinement on *F*^2^	Primary atom site location: structure-invariant direct methods
Least-squares matrix: full	Secondary atom site location: difference Fourier map
*R*[*F*^2^ > 2σ(*F*^2^)] = 0.047	Hydrogen site location: inferred from neighbouring sites
*wR*(*F*^2^) = 0.135	H-atom parameters constrained
*S* = 1.08	*w* = 1/[σ^2^(*F*_o_^2^) + (0.0707*P*)^2^ + 0.2446*P*] where *P* = (*F*_o_^2^ + 2*F*_c_^2^)/3
5872 reflections	(Δ/σ)_max_ < 0.001
228 parameters	Δρ_max_ = 0.40 e Å^−^^3^
0 restraints	Δρ_min_ = −0.23 e Å^−^^3^

### Special details

**Table:**

Geometry. Bond distances, angles etc. have been calculated using the rounded fractional coordinates. All su's are estimated from the variances of the (full) variance-covariance matrix. The cell esds are taken into account in the estimation of distances, angles and torsion angles

### Fractional atomic coordinates and isotropic or equivalent isotropic displacement parameters (Å^2^)

**Table:**

	*x*	*y*	*z*	*U*_iso_*/*U*_eq_	
N1	0.83448 (13)	0.39409 (2)	0.82389 (9)	0.0218 (2)	
C1	0.89994 (14)	0.35082 (3)	0.85268 (9)	0.0180 (2)	
C2	1.05726 (15)	0.33134 (3)	0.79588 (10)	0.0198 (2)	
C3	1.11516 (15)	0.28751 (3)	0.82171 (10)	0.0199 (2)	
C4	1.01916 (14)	0.26098 (3)	0.90243 (9)	0.0187 (2)	
C5	0.86753 (15)	0.28065 (3)	0.96166 (10)	0.0201 (2)	
C6	0.80935 (15)	0.32448 (3)	0.93886 (10)	0.0197 (2)	
C7	1.07388 (14)	0.21366 (3)	0.92100 (9)	0.0184 (2)	
C8	1.30833 (15)	0.19960 (3)	0.99985 (10)	0.0207 (2)	
C9	1.36781 (15)	0.15583 (3)	1.01804 (10)	0.0214 (2)	
C10	1.19330 (14)	0.12377 (3)	0.95599 (9)	0.0193 (2)	
C11	1.24827 (15)	0.07810 (3)	0.97107 (10)	0.0222 (2)	
C12	1.07813 (16)	0.04746 (3)	0.90639 (10)	0.0226 (2)	
C13	0.83518 (15)	0.05968 (3)	0.81869 (10)	0.0207 (2)	
C14	0.65740 (17)	0.02878 (3)	0.74593 (11)	0.0250 (2)	
C15	0.42488 (17)	0.04181 (3)	0.66041 (11)	0.0266 (2)	
C16	0.36320 (16)	0.08558 (3)	0.64636 (10)	0.0236 (2)	
C17	0.53509 (15)	0.11768 (3)	0.71636 (9)	0.0194 (2)	
C18	0.77440 (14)	0.10477 (3)	0.80332 (9)	0.0184 (2)	
C19	0.95341 (14)	0.13704 (3)	0.87271 (9)	0.0176 (2)	
C20	0.89286 (14)	0.18211 (3)	0.85573 (9)	0.0176 (2)	
C21	0.64894 (14)	0.19381 (3)	0.76703 (10)	0.0193 (2)	
C22	0.47894 (14)	0.16319 (3)	0.70109 (10)	0.0204 (2)	
C23	0.94074 (16)	0.42139 (3)	0.74417 (11)	0.0248 (2)	
C24	0.67405 (16)	0.41312 (3)	0.88441 (11)	0.0249 (2)	
H2	1.12420	0.34840	0.73940	0.0240*	
H3	1.22310	0.27520	0.78340	0.0240*	
H5	0.80260	0.26350	1.01910	0.0240*	
H6	0.70730	0.33690	0.98170	0.0240*	
H8	1.43040	0.22070	1.04220	0.0250*	
H9	1.52870	0.14750	1.07320	0.0260*	
H11	1.40770	0.06910	1.02770	0.0270*	
H12	1.12010	0.01750	0.91920	0.0270*	
H14	0.69630	−0.00130	0.75520	0.0300*	
H15	0.30680	0.02050	0.61100	0.0320*	
H16	0.20290	0.09390	0.58870	0.0280*	
H21	0.60590	0.22370	0.75430	0.0230*	
H22	0.31970	0.17210	0.64370	0.0240*	
H52A	0.90310	0.40970	0.64090	0.0370*	
H52B	1.11360	0.42200	0.80130	0.0370*	
H52C	0.87760	0.45110	0.73620	0.0370*	
H52D	0.63160	0.44270	0.84360	0.0370*	
H52E	0.75170	0.41430	0.99680	0.0370*	
H52F	0.52990	0.39530	0.85400	0.0370*	

### Atomic displacement parameters (Å^2^)

**Table:**

	*U*^11^	*U*^22^	*U*^33^	*U*^12^	*U*^13^	*U*^23^
N1	0.0259 (3)	0.0181 (3)	0.0254 (4)	0.0010 (3)	0.0143 (3)	0.0023 (3)
C1	0.0195 (3)	0.0180 (4)	0.0158 (3)	−0.0010 (3)	0.0064 (3)	−0.0002 (3)
C2	0.0232 (3)	0.0206 (4)	0.0181 (3)	−0.0012 (3)	0.0109 (3)	0.0008 (3)
C3	0.0229 (3)	0.0208 (4)	0.0188 (4)	−0.0002 (3)	0.0112 (3)	−0.0007 (3)
C4	0.0211 (3)	0.0185 (4)	0.0169 (3)	−0.0010 (3)	0.0080 (3)	−0.0008 (3)
C5	0.0242 (4)	0.0194 (4)	0.0198 (4)	−0.0015 (3)	0.0121 (3)	0.0004 (3)
C6	0.0226 (3)	0.0196 (4)	0.0197 (4)	0.0001 (3)	0.0114 (3)	0.0000 (3)
C7	0.0219 (3)	0.0189 (4)	0.0159 (3)	−0.0004 (3)	0.0092 (3)	−0.0006 (3)
C8	0.0210 (3)	0.0224 (4)	0.0186 (4)	−0.0012 (3)	0.0079 (3)	−0.0019 (3)
C9	0.0204 (3)	0.0245 (4)	0.0186 (3)	0.0019 (3)	0.0070 (3)	−0.0001 (3)
C10	0.0218 (3)	0.0210 (4)	0.0155 (3)	0.0023 (3)	0.0077 (3)	0.0008 (3)
C11	0.0244 (4)	0.0226 (4)	0.0194 (4)	0.0045 (3)	0.0085 (3)	0.0018 (3)
C12	0.0293 (4)	0.0192 (4)	0.0201 (4)	0.0043 (3)	0.0107 (3)	0.0016 (3)
C13	0.0271 (4)	0.0188 (4)	0.0168 (3)	0.0007 (3)	0.0092 (3)	0.0004 (3)
C14	0.0319 (4)	0.0184 (4)	0.0239 (4)	−0.0022 (3)	0.0103 (3)	−0.0006 (3)
C15	0.0300 (4)	0.0219 (4)	0.0251 (4)	−0.0054 (3)	0.0079 (3)	−0.0012 (3)
C16	0.0239 (4)	0.0233 (4)	0.0215 (4)	−0.0025 (3)	0.0067 (3)	0.0001 (3)
C17	0.0224 (4)	0.0201 (4)	0.0157 (3)	−0.0006 (3)	0.0076 (3)	0.0005 (3)
C18	0.0225 (3)	0.0185 (4)	0.0145 (3)	0.0001 (3)	0.0078 (3)	0.0003 (3)
C19	0.0216 (3)	0.0180 (4)	0.0139 (3)	0.0011 (3)	0.0079 (3)	0.0002 (3)
C20	0.0213 (3)	0.0183 (4)	0.0143 (3)	0.0009 (3)	0.0082 (3)	0.0005 (3)
C21	0.0219 (3)	0.0189 (4)	0.0170 (3)	0.0023 (3)	0.0077 (3)	0.0014 (3)
C22	0.0210 (3)	0.0218 (4)	0.0177 (3)	0.0016 (3)	0.0070 (3)	0.0014 (3)
C23	0.0285 (4)	0.0222 (4)	0.0263 (4)	0.0006 (3)	0.0138 (3)	0.0056 (3)
C24	0.0290 (4)	0.0222 (4)	0.0269 (4)	0.0045 (3)	0.0148 (3)	0.0023 (3)

### Geometric parameters (Å, º)

**Table:**

N1—C1	1.3826 (12)	C17—C22	1.4318 (14)
N1—C23	1.4445 (13)	C18—C19	1.4298 (13)
N1—C24	1.4433 (14)	C19—C20	1.4244 (14)
C1—C2	1.4098 (14)	C20—C21	1.4399 (14)
C1—C6	1.4099 (14)	C21—C22	1.3573 (14)
C2—C3	1.3878 (14)	C2—H2	0.9500
C3—C4	1.3967 (14)	C3—H3	0.9500
C4—C5	1.3986 (14)	C5—H5	0.9500
C4—C7	1.4850 (14)	C6—H6	0.9500
C5—C6	1.3862 (14)	C8—H8	0.9500
C7—C8	1.3990 (14)	C9—H9	0.9500
C7—C20	1.4164 (14)	C11—H11	0.9500
C8—C9	1.3845 (14)	C12—H12	0.9500
C9—C10	1.3995 (14)	C14—H14	0.9500
C10—C11	1.4352 (14)	C15—H15	0.9500
C10—C19	1.4246 (13)	C16—H16	0.9500
C11—C12	1.3565 (14)	C21—H21	0.9500
C12—C13	1.4375 (15)	C22—H22	0.9500
C13—C14	1.4020 (14)	C23—H52A	0.9800
C13—C18	1.4253 (14)	C23—H52B	0.9800
C14—C15	1.3897 (16)	C23—H52C	0.9800
C15—C16	1.3873 (14)	C24—H52D	0.9800
C16—C17	1.4020 (14)	C24—H52E	0.9800
C17—C18	1.4241 (14)	C24—H52F	0.9800
			
C1—N1—C23	120.30 (8)	C20—C21—C22	121.72 (9)
C1—N1—C24	120.01 (8)	C17—C22—C21	121.26 (9)
C23—N1—C24	119.50 (7)	C1—C2—H2	120.00
N1—C1—C2	121.37 (8)	C3—C2—H2	120.00
N1—C1—C6	120.99 (9)	C2—C3—H3	119.00
C2—C1—C6	117.64 (9)	C4—C3—H3	119.00
C1—C2—C3	120.61 (9)	C4—C5—H5	119.00
C2—C3—C4	121.86 (9)	C6—C5—H5	119.00
C3—C4—C5	117.34 (9)	C1—C6—H6	120.00
C3—C4—C7	120.70 (8)	C5—C6—H6	120.00
C5—C4—C7	121.95 (8)	C7—C8—H8	119.00
C4—C5—C6	121.79 (9)	C9—C8—H8	119.00
C1—C6—C5	120.70 (9)	C8—C9—H9	120.00
C4—C7—C8	120.05 (8)	C10—C9—H9	120.00
C4—C7—C20	121.04 (8)	C10—C11—H11	119.00
C8—C7—C20	118.88 (9)	C12—C11—H11	119.00
C7—C8—C9	121.98 (9)	C11—C12—H12	120.00
C8—C9—C10	120.67 (9)	C13—C12—H12	120.00
C9—C10—C11	122.31 (9)	C13—C14—H14	120.00
C9—C10—C19	118.72 (9)	C15—C14—H14	120.00
C11—C10—C19	118.96 (8)	C14—C15—H15	120.00
C10—C11—C12	121.59 (9)	C16—C15—H15	120.00
C11—C12—C13	120.98 (9)	C15—C16—H16	120.00
C12—C13—C14	122.18 (9)	C17—C16—H16	120.00
C12—C13—C18	118.76 (8)	C20—C21—H21	119.00
C14—C13—C18	119.06 (9)	C22—C21—H21	119.00
C13—C14—C15	120.62 (9)	C17—C22—H22	119.00
C14—C15—C16	120.79 (9)	C21—C22—H22	119.00
C15—C16—C17	120.65 (9)	N1—C23—H52A	109.00
C16—C17—C18	119.11 (9)	N1—C23—H52B	110.00
C16—C17—C22	122.15 (9)	N1—C23—H52C	109.00
C18—C17—C22	118.74 (8)	H52A—C23—H52B	109.00
C13—C18—C17	119.77 (8)	H52A—C23—H52C	109.00
C13—C18—C19	120.24 (8)	H52B—C23—H52C	109.00
C17—C18—C19	119.98 (8)	N1—C24—H52D	109.00
C10—C19—C18	119.46 (8)	N1—C24—H52E	109.00
C10—C19—C20	120.36 (8)	N1—C24—H52F	109.00
C18—C19—C20	120.17 (8)	H52D—C24—H52E	109.00
C7—C20—C19	119.37 (8)	H52D—C24—H52F	109.00
C7—C20—C21	122.44 (9)	H52E—C24—H52F	109.00
C19—C20—C21	118.14 (8)		
			
C23—N1—C1—C2	−4.74 (13)	C11—C10—C19—C20	179.94 (8)
C23—N1—C1—C6	175.91 (8)	C10—C11—C12—C13	0.52 (14)
C24—N1—C1—C2	−179.66 (8)	C11—C12—C13—C14	177.63 (9)
C24—N1—C1—C6	0.99 (13)	C11—C12—C13—C18	−1.31 (14)
N1—C1—C2—C3	−177.93 (8)	C12—C13—C14—C15	−179.17 (9)
C6—C1—C2—C3	1.44 (13)	C18—C13—C14—C15	−0.23 (14)
N1—C1—C6—C5	177.17 (9)	C12—C13—C18—C17	179.69 (8)
C2—C1—C6—C5	−2.20 (13)	C12—C13—C18—C19	0.73 (13)
C1—C2—C3—C4	0.79 (14)	C14—C13—C18—C17	0.72 (13)
C2—C3—C4—C5	−2.22 (13)	C14—C13—C18—C19	−178.25 (9)
C2—C3—C4—C7	176.62 (8)	C13—C14—C15—C16	−0.61 (15)
C3—C4—C5—C6	1.44 (13)	C14—C15—C16—C17	0.95 (15)
C7—C4—C5—C6	−177.39 (8)	C15—C16—C17—C18	−0.44 (13)
C3—C4—C7—C8	62.00 (11)	C15—C16—C17—C22	178.41 (9)
C3—C4—C7—C20	−115.98 (10)	C16—C17—C18—C13	−0.39 (13)
C5—C4—C7—C8	−119.22 (10)	C16—C17—C18—C19	178.58 (8)
C5—C4—C7—C20	62.80 (11)	C22—C17—C18—C13	−179.28 (8)
C4—C5—C6—C1	0.77 (14)	C22—C17—C18—C19	−0.31 (12)
C4—C7—C8—C9	−179.10 (8)	C16—C17—C22—C21	−178.57 (9)
C20—C7—C8—C9	−1.07 (13)	C18—C17—C22—C21	0.30 (13)
C4—C7—C20—C19	178.40 (8)	C13—C18—C19—C10	0.63 (12)
C4—C7—C20—C21	0.82 (13)	C13—C18—C19—C20	179.27 (8)
C8—C7—C20—C19	0.39 (12)	C17—C18—C19—C10	−178.34 (8)
C8—C7—C20—C21	−177.19 (8)	C17—C18—C19—C20	0.30 (12)
C7—C8—C9—C10	0.64 (14)	C10—C19—C20—C7	0.69 (12)
C8—C9—C10—C11	179.36 (9)	C10—C19—C20—C21	178.38 (8)
C8—C9—C10—C19	0.47 (13)	C18—C19—C20—C7	−177.94 (8)
C9—C10—C11—C12	−178.03 (9)	C18—C19—C20—C21	−0.26 (12)
C19—C10—C11—C12	0.86 (13)	C7—C20—C21—C22	177.84 (9)
C9—C10—C19—C18	177.52 (8)	C19—C20—C21—C22	0.24 (13)
C9—C10—C19—C20	−1.12 (12)	C20—C21—C22—C17	−0.26 (14)
C11—C10—C19—C18	−1.41 (12)		

### Hydrogen-bond geometry (Å, º)

Cg1, Cg2, Cg3, Cg4 and Cg5 are the centroids of the C1–C6, C7–C10/C19/C20, C10–C13/C18/C19, C13–C18 and C17–C22 rings, respectively.

**Table:**

*D*—H···*A*	*D*—H	H···*A*	*D*···*A*	*D*—H···*A*
C2—H2···*Cg*2^i^	0.95	2.93	3.6043 (15)	129
C6—H6···*Cg*5^ii^	0.95	2.90	3.6495 (15)	137
C22—H22···*Cg*1^iii^	0.95	2.68	3.5499 (15)	152
C23—H52*A*···*Cg*3^i^	0.98	2.74	3.5994 (15)	147
C24—H52*E*···*Cg*4^ii^	0.98	2.77	3.5284 (15)	135

Symmetry codes: (i) *x*, −*y*+1/2, *z*−1/2; (ii) *x*, −*y*+1/2, *z*+1/2; (iii) *x*−1, −*y*+1/2, *z*−1/2.
